# Design and evaluation of a positive intervention to cultivate mental health: preliminary findings

**DOI:** 10.1186/s41155-021-00172-1

**Published:** 2021-02-24

**Authors:** Susana Gorbeña, Leila Govillard, Ignacio Gómez, Sare Sarrionandia, Patricia Macía, Patricia Penas, Ioseba Iraurgi

**Affiliations:** 1 Departamento de Personalidad, Evaluación y Tratamientos Psicológicos, Facultad de Psicología y Educación, Universidad de Deusto, Apartado 1, 48080 Bilbao, Spain; 2 Departamento de Trabajo Social y Sociología, Facultad de Ciencias Sociales y Humanas, Universidad de Deusto, Camino de Mundaiz 5, 20012 San Sebastián, Spain; 3 Departamento de Psicología Social y del Desarrollo, Facultad de Psicología y Educación, Universidad de Deusto, Apartado 1, 48080 Bilbao, Spain

**Keywords:** Positive manualized intervention, Positive mental health, Well-being, Flourishing, Pilot test

## Abstract

The past two decades have witnessed a proliferation of positive psychological interventions for clinical and non-clinical populations, and recent research, including meta-analyses, is providing evidence of its effectiveness. Most interventions have focused on increasing life satisfaction, positive affect, and psychological well-being. Manualized, multi-component interventions based on a comprehensive theory are scarce. Keyes’ concept of mental health and flourishing (subjective, psychological, and social well-being) is an overarching theoretical framework to guide the design of a multi-component psychological intervention to cultivate well-being and personal development. Therefore, the purpose of this study was to design a theory-driven positive intervention and to pilot test the intervention. The manual presents an 8-week group program that includes homework activities. A sample of 56 young adults completed the intervention. Participants were assessed at base line, after termination, and at a 6-month follow-up session. Standardized instruments were used to assess the dimensions of mental health proposed by Keyes. Pre- and post-test measures of subjective, psychological, and social well-being showed significant differences, as did the total mental health scores. At 6-month follow-up, differences remained in subjective and psychological well-being and in positive mental health, with smaller effect sizes. Limitations of these preliminary findings as well as future lines of research and improvements in this manualized intervention are proposed in the light of current research on positive interventions.

## Introduction

Following Seligman et al.’s (2005) publication providing the first evidence of the effects of positive psychological interventions (PPIs), researchers and practitioners started to develop and assess a variety of these interventions designed to increase positive affect, well-being, optimism, personal strengths, hope, and self-esteem, as well as to decrease clinical symptoms of distress. Several meta-analyses and systematic reviews were published in the past 12 years, providing information about the effectiveness of these interventions. Sin and Lyubomirsky (2009) reviewed 49 studies, utilizing primarily non-clinical samples and concluded that PPIs were effective in increasing well-being and reducing depression. Bolier et al. (2013) replicated this finding in a meta-analysis examining 39 randomized controlled studies. A recent review (Chakhssi et al., 2018), focusing on diverse clinical samples, also concluded that significant, though small to moderate effect sizes, were found for well-being, depression, and anxiety. However, according to Ghosh and Deb (2017), the effectiveness of PPIs with individuals with chronic physical illnesses is inconclusive, since half of the studies included in their meta-analysis reported insignificant findings. The latest and most comprehensive meta-analysis, by Koydemir et al. (2020), also concluded that PPIs display promising effectiveness for increasing well-being in non-clinical adult populations.

Despite this propitious scenario for PPIs, their diversity in terms of goals, target populations, contents, delivery format, and duration yields a complex picture (Bolier et al., 2013). For instance, published studies present differences in terms of goals and outcome measures, with interventions targeting different dimensions of well-being, mostly subjective well-being and psychological well-being (Koydemir et al., 2020; Weiss, et al., 2016) or focusing in different psychological constructs such as optimism, hope, or happiness. Furthermore, another major goal has been the alleviation of depressive symptoms, a work that Fava initiated in the 90s with his Well-Being Therapy (Fava, 1999). In sum, this diversity of goals and outcome measures hinders sound conclusions about the effectiveness of PPIs.

Studies examining PPIs have utilized different populations in terms of age and health conditions (Bolier et al., 2013; Koydemir et al., 2020). For instance, there are interventions specifically designed for individuals with either mental or physical health problems, with much debate occurring in the area of cancer patients. There is also diversity in terms of the specific content and activities proposed. Many single component interventions present only one activity (see Stone and Parks, 2018, for a review of the most popular and well-researched interventions), whereas others, known as multicomponent interventions focus on different topics and propose a variety of activities. Two examples are Fordyce’s (1977) Happiness Program, and Seligman’s Positive Psychotherapy Program (Seligman et al., 2006). As its regards to format, interventions are delivered individually, in groups, on-line, or are self-administered. Sin and Lyubomirsky (2009) found in their meta-analysis that the most effective modality was individual followed by group interventions, but Bolier et al. (2013) did not. The literature also addresses the issue of duration. According to Sin and Lyubomirsky (2009), Koydemir et al. (2020), and Bolier et al. (2013), longer interventions (8 to 12 weeks) produced greater gains in well-being.

Finally yet importantly, several voices have raised the issue of the lack of a unifying conceptual framework (Parks & Biswas-Diener, 2013; Wong & Roy, 2018) and the fact that many interventions were designed without a background theory, a question of paramount importance in psychological interventions. In sum, PPIs constitute a new development in the treatment arena that can become efficient tools to improve well-being and mental health.

The analysis of the available literature on PPIs leads the authors to conclude that the major limitation is the lack of a theoretical framework. However, in recent years, some models have been advanced. Keyes’ (2002, 2003, 2005) theory of mental health is especially suitable to inform positive interventions. He described mental health as something different from the mere absence of mental illness. Mental health is operationalized as a syndrome of symptoms of positive feelings and positive psychosocial functioning. Keyes coined the term positive mental health or flourishing and analyzed, using a large US population sample, its relationship with mental illness and other psychological variables and life outcomes (Keyes, 2005; Keyes et al., 2010). According to Keyes, the three components of mental health are subjective or emotional well-being, psychological well-being, and social well-being. Subjective well-being refers to the presence of positive emotions and life satisfaction. Following Ryff (1989), psychological well-being is characterized by high levels of self-acceptance, positive relations with others, personal growth, purpose in life, environmental mastery, and personal autonomy. Finally, Keyes stated that positive functioning also encompasses social well-being, a dimension of mental health that includes social coherence, social actualization, social integration, social acceptance, and social contribution (Keyes, 1998). Keyes defined mental health or flourishing, as a state characterized by high levels of well-being, “a state in which an individual feels positive emotion toward life and is functioning well psychologically and socially” (Keyes, 2003, p. 294). Its opposite was termed languishing.

Despite the fact that Keyes has suggested the application of this theory to guide interventions (Keyes, 2003, 2007; Keyes & Lopez, 2002), to our knowledge, no intervention has been proposed applying this theoretical framework. A review of PPIs showed that some have used, as an outcome measure, the construct of mental health or flourishing, but these interventions were not developed with such framework in mind. In an attempt to palliate this lack of theoretical foundation in the PPIs arena, the main goal of this study was to design a theoretically driven intervention to promote positive mental health or flourishing, and to pilot test this intervention.

## Method

### Participants

Participants were college students recruited at a medium-size university. The center offered a workshop on well-being and personal development as an extracurricular activity. Fourteen individuals from different majors enrolled and completed the program in two separate groups. Besides, 43 participants of an elective course in Psychology voluntarily participated in the workshop. Two persons declined participation. There were no differences in terms of age, gender, and major outcome variable (positive mental health) in these groups and therefore they were added to form the final sample of 57 individuals for this study. Age ranged from 18 to 44 with a mean of 21.8 (SD = 3.8). All were Caucasian, and 89.5% were females. Preliminary analysis identified, at base line and post-test, a case with a pattern of response clearly polarized to the extremes that was considered as an outlier and eliminated from the final analysis. Finally, response rate at 6-month follow-up was 79% of the initial sample, 44 participants, with no significant differences in the outcome variables between present and lost cases, both in base line and post-test.

### Instruments

The instruments used to obtain a measure of positive mental health that participants completed at base line, post-test, and 6 month follow-up were the following.

Satisfaction with life scale-SWLS (Diener et al., 1985) adapted to Spanish by Vázquez et al. in 2013. It consists of five items with a seven point format response from “*strongly agree*” to “*strongly disagree*.” It is a sound and widely used measure and internal consistency in the Spanish adaptation was .88. In this study, the alpha was .82.

Scale of Positive and Negative Experience (SPANE) developed by Diener et al. (2010) as a new measure of the amount of time positive and negative emotions are experienced in the past four weeks. It includes 12 items with a response format ranging from one (*very rarely or never*) to five (*very often or always*). It yields three scores: positive, negative, and balance affect but only the positive affect score was used in this study. Cronbach alpha for the positive affect scale was .87 in the original study, .86 in a Spanish adaptation (Espejo et al., 2020), and .83 in the present sample.

Psychological Well-being scales (Ryff, 1989) adapted to Spanish (Díaz et al., 2006). This version consisted of 39 items with a 6-point Likert scale response format, from “*strongly agree*” to “*strongly disagree*.” The scales measure self-acceptance, positive relations with others, autonomy, environmental mastery, purpose in life, and personal growth. It has been widely used in the literature and it is considered a sound measure of positive functioning in the eudaimonic tradition of well-being research (Keyes, 2013; McDowell, 2010). The alpha in the present study was .88.

Social well-being was measured using the Spanish adaptation of Keyes’ instrument (Keyes, 1998) published by Blanco and Díaz in 2005. It measures five dimensions of social well-being: integration, acceptance, contribution, coherence, and actualization, and it consists of 33 items, with a 5-point response format, from “*strongly agree*” to “*strongly disagree*.” The Spanish adaptation eliminated eight items but, given that it was done with a small non-representative sample, we decided to maintain all the items of Keyes’ original scale. Cronbach’ alpha obtained in this study for the total scale was .89.

### Procedure

Procedures are described in two separate sections: those referring to the design of the intervention, and those involved in the implementation of the program. In terms of design, after conducting a thorough review of the literature, the authors made decisions about topics and methodology based Keyes’s construct of positive mental health or flourishing. We followed two criteria to select topics and activities. First, the program had to include content related to the three dimensions of well-being that form the construct of positive mental health or flourishing. Second, when possible, we choose activities previously tested. However, as it is mentioned below, some new activities were proposed, especially to cover the social well-being dimension. The program consisted of eight sessions of 1 h and 40 min, plus the base line and follow-up sessions. Each session presented a different topic using a combination of group or dyadic dialog, brief presentations, audiovisual material, exercises and tests, and testimonies. One-third of the sessions was devoted to reviewing the homework assignment of the previous week. Participants in the two extracurricular activity groups worked with one facilitator in groups of seven people Participants of the elective course worked with four facilitators in groups of 10–11 individuals. Participants received a folder to file the materials, and a *Well*-*being Notebook* to take notes and complete the homework. A Google site was available with all the materials used.

A brief description of main topics and homework assignments in each session follows, with a summary presented in Table 1.

**Table 1:** Well-being and personal development program: summary of sessions

Session title	Main topics	Homework assignment
Well-being and personal development	Expectation, norms and commitments Personal beliefs about happiness, well-being and personal development Well-being incremental mind-set	Identifying a personal source of well-being
Positive emotions	Positive and negative emotions Functionality of positive emotions Strategies to cultivate positive emotions	Three good things
Personal and social gratitude	Meaning of gratitude Gratitude to society Planning the gratitude letter and visit	Thanks Europe! Gratitude letter and visit
Personal strengths	Personal strengths: insights and VIA test	Checking my strengths with significant others
Meaning and purpose in life	Diversity of meanings and purposes Reflection on personal life meaning	Taking meaningful pictures
Best possible self and world	Imagery of best professional future self Subjective vs objective world view: Factfulness test	Best possible self and world
Personal growth	Personal growth initiative The comfort zone	Out of my comfort zone
Summary	Compilation of learning and experiences Group creative activity Farewell	Final essay

#### Introduction

The first session worked on expectations and beliefs about happiness, well-being, and personal development using instruments developed by McMahan and Estes (2011), and Zacarés and Serra (1996). Time was also devoted to clarify the rules of the group and to begin to develop group cohesion. Following Howel, et al. (2016) we worked to facilitate a growth mind-set about well-being and personal development. We also discussed basic concepts of well-being and mental health (Keyes, 2002). The first session also introduced the role and importance of homework throughout the experience. There was no formal control of its performance but facilitators highlighted the expected benefits of practicing the proposed activities and the commitment with the group work. The first homework assignment asked participants to select, reflect, and bring an example of a source of personal well-being, like a picture, a preferred leisure activity, a significant other, an experience, a personal resource, or the like.

#### Second session

The session begins encouraging participants to share their homework with the group, in this case personal examples of sources of well-being. Individuals thus become aware of the diversity of sources of well-being and gather ideas to apply to their own lives. Then, the session focuses on positive and negative emotions. We followed Fredrickson (2003) and Lyubomirsky (2007) to discuss their functionality, and offered suggestions on how to manage negative emotions and cultivate positive ones. Illustrations and audiovisual materials helped participants to gather ideas to work on this issue. Finally, Seligman’s activity *Three good things* (Seligman et al., 2005) was presented and proposed as a homework activity for the week. It asks individuals to keep a daily log of three experiences during the day that elicited a positive emotion. At the end of the week, and before the next session, participants were encouraged to review the log and draw some personal conclusions.

#### Third session

Once participants had discussed their gains doing *Three good things* during the week, a group discussion on the meaning, reasons, and recipients of gratitude was conducted. Using a section of a movie, awareness of social gains and the benefits of the Welfare State in Europe was facilitated, so that gratitude to our society and to the efforts and struggles of previous generations could be elicited. Time was devoted to thoroughly explain and plan the *Gratitude Letter and Visit* (Seligman et al., 2005). The activity proposes participants to identify a person in their lives they feel gratitude toward and had not expressed it adequately. Then they are asked to write a letter to that person and deliver the content in a personal encounter. The weekly homework also included finishing and reflecting on movie seen in the session.

#### Fourth session

This session first reviewed accomplishments with the letter and visit, and insights from the movie. It later moved to personal strengths asking people to identify and share in dyads their strengths. A short version of the VIA (*Values in Action Inventory of Strengths*) (Peterson & Seligman, 2004) was used. The VIA is an instrument that explores 24 personal strengths grouped in six families of human virtues and yields an output identifying the five most salient strengths of the person. Participants discussed in small groups of two to three people the degree of agreement and concordance between the test and personal insights. Homework asked participants to ask three significant others about the strengths they thought they had, and to reflect on the differences and similarities of these three different sources of information on strengths.

#### Fifth session

After reviewing homework assignments, the session focused on the exploration of the variety of meanings and purposes in life. Video materials as well as quotes from famous people were used to elicit personal and group insights. Steger et al. (2013) designed an activity to facilitate personal exploration of life’s meaning. It is a photographic method that asks individuals to take several photographs of things that make life meaningful to them. Participants were asked to take, bring, and reflect on pictures that represented the sources of meaning in their lives.

#### Sixth session

Upon sharing and explaining sources of meaning and purpose using the photographs taken during the previous week, this session was devoted to goal directed behavior and future expectations. Following work on best possible self (King, 2001), an imagery exercise was proposed to explore one’s best professional self. The facilitator guided participants to envision their professional life in 10 years and to elaborate in detail the characteristics of this situation. They later wrote in the *Well*-*Being Notebook* what they had imagined. Furthermore, to cultivate the social well-being dimensions, we designed a novel activity applying the best possible self-methodology to the social arena. Using ideas from Rosling et al. (2018) and his *Factfulness test*, insights were facilitated about the positive evolution of humankind, and discussion was encouraged about what world participants would want to live in 10 years ahead. Individuals gained, using worldwide historical facts generated by Rosling, a new understanding of the evolution of society and the accomplishments of humankind. Finally, the homework requested participants to work on the other life domains of the best possible self-activity such as interpersonal, family and intimate relationships, leisure, or social participation. They also had to articulate what were some of the features of the world and society they would like to live in the future.

#### Seventh session

This session first gave participants the opportunity to share their homework and feelings about it. The new topic was personal growth and development, and was facilitated using audiovisual material about getting outside of one’s comfort zone. Thoen and Robitschek’s (2012) *Manual of Intentional Growth Training* was adapted and used. Subjects were encouraged to plan and start performing a new and desired behavior, out of their comfort zone, they felt will lead to personal growth.

#### Eight session

The final session reviewed participants’ accomplishments with the last homework assignment. To summarize the experience and learning, we invited participants to do creative group activity to reflect on the program in terms of their individual and group experience. Groups created a joint collage using diverse material, presented it, and finished taking some pictures. As a final homework assignment, they were asked to write an open essay about the experience.

As mentioned above, each session and activity was hypothesized to cultivate one or more of the dimensions of positive mental health or flourishing. A major challenge was to include topics and exercises related to social well-being. As it has been described, several strategies were used to achieve this objective. For instance, use of a group format for program delivery, emphasis on social diversity as it regards to the different topics covered, work on social gratitude, exploration and integration of views of others about self, awareness and reflection on the role of our physical and social context at both micro and macro levels, and articulation of best possible future world.

Finally, the program included one more session to gather the post intervention data and assess satisfaction with the workshop.

As it regards to implementation, first, the project secured the approval of the Research Ethic Board of the university. All participants signed an informed consent and received a numerical code for identification to dissociate personal data. Participants were randomly assigned to the groups. The program was implemented during the second semester of the school year, avoiding coincidence with school holidays and exam periods. Finally, after 6 months, participants were contacted via electronic mail and an on-line questionnaire was administered.

Trained psychologists, under the supervision of a certified clinical psychologist, conducted the sessions. Following Worth (2017), a training program for the facilitators was implemented (also described in detail in the manual) that included reading materials, personal reflection on the topics, group discussions, and personal experience with all the homework activities. Each session was prepared in a group meeting. Furthermore, after each session, a supervision encounter took place. Facilitators were also asked to keep and share in supervision a diary of each session and signed a confidentiality contract.

### Data analyses

Three outcome indicators (subjective well-being, psychological well-being, and social well-being) were created from the specific questionnaires of each construct and following the indications of the authors who designed them. The positive mental health indicator was created calculating the average sum of the three well-being indicators. The indicators were transformed into a decimal scale from the following algorithm: (*∑x*_*i*_ *–* min * 10/max), being *∑x*_*i*_ the sum of the scores of the items that compose the instruments, and where min and max are the minimum and maximum values of the possible range.

We calculated the mean (M) and standard deviation (SD) for the three assessment times, and the normality of the distributions was verified with the Kolmogorov-Smirnov test. Since this is a repeated measures factor design, the spherical assumption of the variance-covariance matrix was checked with the Mauchly’s *W* test, and the analysis of variance *F* test was used through the 'general linear model—repeated measures’ routine of the SPSS-v24 program. This same routine provides *F* tests for the polynomial contrasts (linear and quadratic trend), the effect sizes through the partial eta coefficient (*η*^2^), and the statistical power of the test (1-*β*); all of them for each of the *F* tests used. Similarly, this routine provides the probability values (*p*) of the comparisons between pairs of time measures (T1-T2, T1-T3, and T2-T3). Finally, in order to compare differences in means in T1-T2, and T1-T3, a *t* test for paired samples was used, calculating the mean (M_dif_) and the standard deviation (SD_dif_) of the differences, along with the 95% confidence interval (CI 95%). Finally, effect sizes of the differences between T1 and T2 and T1 and T3 were calculated using Cohen’s *d* for paired samples, as suggested by Lakens (2013). A commonly used interpretation is to refer to effect sizes as small (*d* = 0.2), medium (*d* = 0.5), and large (*d* = 0.8) based on benchmarks suggested by Cohen (1988). However, these values are arbitrary and should not be interpreted rigidly (Thompson, 2007). Therefore, the best way to interpret Cohen’s *d* is to relate it to other effects found in in the relevant literature.

The datasets used during the current study are available from the corresponding author on reasonable request.

## Results

Table 2 presents descriptive statistics at pre, post-test, and 6-month follow-up of all outcome variables. High scores were found in the variables in the three moments, with the highest scores after the intervention (T2). Table 2 also reports the tests of normality for all distributions, and in all cases the probability values associated with the Kolmogorov-Smirnov test have been statistically non-significant (*p* > .05), not being able to reject the null hypothesis and, therefore, assuming the normality of the distributions. The two columns to the right of Table 2 offer results of the Mauchly’s sphericity test, which, in all cases, the probability value associated with the *W* statistic was greater than the critical level (*p* > .05) and, therefore, it is assumed that the variance-covariance matrix is spherical.

**Table 2:** Base line, post-test and 6-month measures: means, standard deviations, normalized test (Kolmogorov-Smirnov—K-S) and sphericity test (Mauchly’s *W*)

	T1 (*n* = 56)		T2 (*n* = 56)		T3 (*n* = 44)		Sphericity test	
	M (SD)	*K-S*	M (SD)	*K-S*	M (SD)	*K-S*	*W*	*p*
Subjective well-being	7.08 (1.19)	.070	7.55 (1.27)	.060	7.37 (1.50)	.055	.979	.635
Psychological well-being	7.01 (0.94)	.064	7.37 (0.89)	> .10	7.29 (1.04)	> .10	.998	.960
Social well-being	5.44 (0.76)	> .10	5.79 (0.68)	> .10	5.40 (0.82)	> .10	.893	.092
Positive mental health	6.51 (0.82)	> .10	6.91 (0.79)	> .10	6.69 (0.99)	> .10	.983	.703

**Fig. 1:**
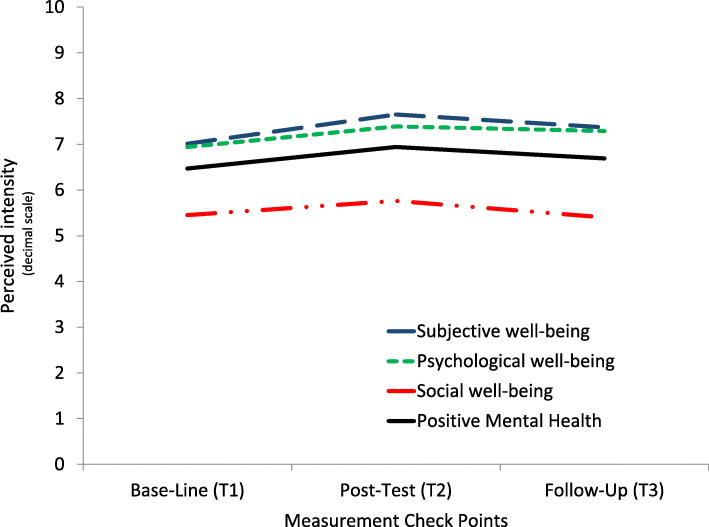
Repeated measures trends of the principal outcomes variables

Once the assumptions of the application of a factor repeated measurements (independence, normality, and sphericity) were checked, we conducted four analyses of variance, whose results are represented together in Table 3 and Fig. 1. For the four indicators evaluated, the overall *F* test has been statistically significant (*F* values for 2 and 86 degrees of freedom greater than 8.66; *p* < .001; Table 3), and it can be concluded that the averages compared are not equal. The effect sizes obtained in this test have been high (*η*^2^ > .168, equivalent to a Cohen’s *d* > .898) and have adequate statistical power (1-*β* > .950). The polynomial contrasts do not allow rejecting the linear component of the trend—except in the case of the social well-being indicator (*F* = 0.32; *p* = .573)—although in all cases a quadratic trend in the form of an inverted V prevails with greater probability. On the other hand, comparisons between pairs offer statistically significant differences in all indicators between baseline and post-test (*p* < .001), and between baseline and follow-up in the case of psychological well-being (*p* = .006) and between post-test and follow-up in the case of the positive mental health indicator (*p* = .05).

**Table 3:** Repeated measures ANOVA for outcome variables

	Global and polynomial contrast												*p* value for temporal comparisons		
	Global				Linear				Quadratic						
	*F*	*p*	*η*^2^	*1*-*β*	*F*	*p*	*η*^2^	*1*-*β*	*F*	*p*	*η*^2^	*1*-*β*	T1-T2	T1-T3	T2-T3
Subjective well-being	8.66	< .001	.17	.96	4.87	.033	.10	.58	13.68	< .001	.24	.95	< .001	.098	.226
Psychological well-being	10.06	< .001	.19	.99	10.67	.002	.20	.89	9.42	.004	.18	.85	< .001	.006	.896
Social well-being	9.48	< .001	.18	.98	0.32	.573	.01	.09	21.58	< .001	.33	.99	< .001	.999	.095
Positive mental health	11.27	< .001	.21	.99	4.52	.039	.09	.55	19.57	< .001	.31	.99	< .001	.118	.050

Notes. F: F test; p: probability value; η^2^: partial eta-square (effect size); 1-β: statistical power

An analysis of the change between baseline and post-test scores (T1–T2), and between baseline and follow-up (T1–T3) is presented in Table 4, with the intention of assessing the effect size achieved. Change observed in all variables after the intervention (comparison T1–T2) has been positive, showing a significant increase in positive mental health, subjective, psychological, and social well-being. Effect sizes for positive mental health (*d* = .50), social well-being (*d* = .48), subjective well-being (*d* = .40), and psychological well-being (*d* = .38) were moderate-low. The differences in follow-up scores with respect to the beginning of the program are positive in the cases of psychological well-being (*M*_*dif*_ = 0.35; *t* = 3.27, *p* = .002, *d* = .35), subjective well-being (*M*_*dif*_ = 0.36; *t* = 2.21, *p* = .033, *d* = .25), and positive mental health (*M*_*dif*_ = 0.21; *t* = 2.13, *p* = .039, *d* = .23). Non-significant changes were observed (*M*_*dif*_ = − 0.05; *t* = − 0.56, *p* = .573, *d* = .06) in the case of social well-being.

**Table 4:** Change assessment between baseline and post-intervention (T1–T2), and between baseline and 6-month follow-up (T1–T3)

	T1–T2 comparisons (*n* = 56)							T1–T3 comparisons (*n* = 44)						
	*M*_*dif*_	*SD*_*dif*_	(Interval 95%)		*t*	*p*	*d*	*M*_*dif*_	*SD*_*dif*_	(Interval 95%)		*t*	*p*	*d*
Subjective well-being	0.48	1.01	(0.21	0.76)	3.59	.001	.40	0.36	1.10	(0.03	0.70)	2.21	.033	.25
Psychological well-being	0.35	0.69	(0.16	0.54)	3.77	.001	.38	0.35	0.70	(0.13	0.56)	3.27	.002	.35
Social well-being	0.35	0.51	(0.21	0.49)	5.12	.001	.48	− 0.05	0.63	(− 0.24	0.14)	− 0.56	.573	.06
Positive mental health	0.40	0.61	(0.23	0.56)	4.84	.001	.50	0.21	0.68	(0.01	0.43)	2.13	.039	.23

*d:* values are *d*_*mr*_ for repeated measures as suggested by Lakens (2013)

## Discussion

The study outcomes show an improvement in self-report measures of positive mental health (subjective, psychological, and social well-being) after the proposed intervention. At 6-month follow up the scores tend to regress to base line measures but are still significant for subjective and psychological well-being, and the overall positive mental health index. Social well-being returned to base line levels. The results for subjective and psychological well-being are consistent with previous research on the effects of PPIs (Bolier et al., 2013; Koydemir et al., 2020). We found only one study (Key-Roberts, 2010) that used social well-being as an outcome measure, with no improvements after the intervention. Perhaps the intervention we designed did not address sufficiently this dimension, or the effects of continuous practice, after the intervention was over, were lost. An alternative explanation could be that other strategies might be more efficient in order to cultivate social well-being. For instance, active involvement in community life, volunteering, and civic participation might be necessary to maintain social well-being. Further research is needed to explore these issues.

Some important limitations of our study must be mentioned. The major limitation is the lack of a control group that prevents us from any assumption of causality. This is a pre experimental study designed to develop a PPI and to conduct an initial testing of the proposal. Therefore, results cannot be attributed to the intervention. The possibility exists that participants might have benefited from the group experience itself. Besides, expectations might have influenced the results, or other uncontrolled variables such as the adherence to the homework assignments. Another important limitation is attributable to the fact that our findings are based on a sample that was not randomly assigned to the intervention but self-selected. The motivation to improve might also explain the results, an issue Sin and Lyubomirsky (2009) pointed out in their review. Another limitation of our study is the characteristics of the sample, a small group of young Caucasian adults, college students, and mostly females. As Hendriks et al. (2018) mention in their conclusions, studies on PPIs should be done using more diverse samples in terms of ethic and cultural background, gender, and age. Finally, an important limitation refers to the fact that is difficult to isolate the contribution of any one topic or activity proposed in the intervention, and thus we are not able to identify elements that facilitate change. Yet, as Parks and Biswas-Diener (2013) mentioned, a multicomponent intervention resembles more real life use of activities by individuals and practitioners. In sum, because of all the above-mentioned limitations, our work should be interpreted with caution as a pilot and preliminary study that nevertheless can spark interest in further research.

Despite the limitations, these preliminary results inspire us to continue the exploration of the potential effects of this intervention. The two main issues to address in future work are the improvement of the intervention program itself, and the realization of rigorous studies to analyze its effectiveness.

The authors have identified at least six changes to improve the manual developed for this intervention. First, we suggest a change in the order of sessions. The gratitude letter and visit is a powerful and, for some individuals, a challenging activity that might be better placed after the work on personal strengths, and thus be presented in the fourth session. Second, following suggestions by Biswas-Diener et al. (2011), work on personal strengths can also be improved. Third, as others have pointed out (Parks & Biswas-Diener, 2013), some activities, such as the best possible self, can be unpleasant for some individuals, as it happened in our experience. In a similar vein, this group of young adults had some difficulty working on life meaning and purpose. Therefore, adaptations or alternatives could be design to be implemented if uneasiness appears. Similarly, the delivery of the program to other groups with different cultural backgrounds or of different age groups demands adaptations. For instance, our current experience with adults and elders suggests that work on life goals through the best possible self-activity might not be relevant for this age group and can be replaced with another activity such as reminiscence, life narratives, or forgiveness, as Durgante and Dell’Aglio (2019), and others have done. Another identified improvement is the inclusion of additional optional homework activities for those individuals interested in a given topic. Fifth, we also suggest that the duration of the sessions be increased in at least 20 min, being of two full hours. Finally, it would be desirable to incorporate some suggestions and resources, as long-term homework, to facilitate the maintenance of gains.

Future studies should test the intervention using different types of control groups and diverse samples. We also have to elucidate the role of some important mediating variables. For instance, it will be important to explore the role of cognitive and motivational variables such as implicit theories of well-being (Howell et al., 2016). The intervention worked on this issue and tried to promote an incremental view of well-being but the lack of a pre-post-test assessment of such variable impedes any conclusions. Besides, future studies should analyze and control other variables to assess the impact of these interventions. Personality traits, coping strategies, self-efficacy, and stress levels, among others, are important elements to understand how mental health can be improved. Another important variable that deserves further analysis is the group and its therapeutic effects (Corsini & Rosenberg, 1955). Finally, the combination of quantitative and qualitative methodologies can also contribute to gain a better understanding of the processes involved in individual growth and change. In fact, Hefferon et al. (2017) made a recent call to increase qualitative research in positive psychology. The final homework assignment of this intervention, an open-ended essay, is in the process of study. It might shed light in terms of the perceived outcomes and the processes involved in positive change.

## Conclusions

There are two major conclusions that can be drawn from this study. First, Keyes’ construct of positive mental health or flourishing can be a useful framework to design a theory-driven multicomponent psychological intervention. Such intervention addresses three areas of positive mental health (subjective, psychological, and social well-being) that are hypothesized to be susceptible to improvement and change. The second conclusion refers to the need to go beyond this exploratory study and design rigorous studies to test the efficacy and effectiveness of the proposed intervention, and to explore the different personal, contextual, and program variables that might mediate or moderate the expected positive outcomes. Participants improved their mental health 6 months after they partaken in the program, but scientific evidence to claim the benefits of this program remains to be presented.

## Data Availability

The datasets used during the current study are available from the corresponding author on reasonable request.
